# Nectin-4 is widely expressed in head and neck squamous cell carcinoma

**DOI:** 10.18632/oncotarget.28299

**Published:** 2022-10-20

**Authors:** Christine Sanders, Jan-Frederic Lau, Dimo Dietrich, Sebastian Strieth, Peter Brossart, Glen Kristiansen

**Affiliations:** ^1^Institute of Pathology, University Hospital Bonn (UKB), Bonn, Germany; ^2^Department of Otorhinolaryngology, University Medical Center Bonn (UKB), Bonn, Germany; ^3^Department of Haemato-oncology, University Hospital Bonn (UKB), Bonn, Germany

**Keywords:** Nectin-4, enfortumab-vedotin, HNSCC, p16

## Abstract

Purpose: Nectin-4 has been successfully established as a target molecule in locally advanced and metastatic bladder cancer. An antibody-drug conjugate (enfortumab-vedotin) directed against nectin-4 has shown marked tumor remission rates in this tumor type, which is known for high expression rates of nectin-4. As head and neck cancer and urothelial carcinomas share morphological and molecular similarities, we aimed to evaluate Nectin-4 expression in head and neck squamous cell carcinoma (HNSCC).

Material and Methods: A previously described and clinically characterized cohort of HNSCC (*n* = 159) was analyzed by immunohistochemistry for Nectin-4 expression. The expression data was correlated to clinico-pathological parameters including patient outcome.

Results: Nectin-4 was found in 86.2% of HNSCC, with medium/high expression seen in 32.7% of cases. Non smokers and p16 positive HNSCC showed a higher expression of Nectin-4 (*p* < 0.005). There was no correlation of Nectin-4 with grading or tumor stage. Nectin-4 positive tumors showed a significant better survival (log rank *p* = 0.006).

Conclusions: Similar to urothelial carcinoma, Nectin-4 is found in the majority of HNSCC, which clearly warrants further studies to clarify if HNSCC also respond to targeted therapy with enfortumab-vedotin. Moreover, expression of Nectin-4 is associated with HPV infection and may serve as a prognostic marker in HNSCC.

## INTRODUCTION

Nectins are immunoglobulin-like cell adhesion molecules and regulate besides cell adhesion various cellular functions, such as cell motility, proliferation, polarization, survival and differentiation [1–3] and appear to have an immunomodulatory function [4, 5]. Four nectins have been described. Nectin-4 has been shown to be a successful therapy target in solid tumors including bladder cancer. Challita-Eid et al. conducted an immunohistochemical analysis of Nectin-4 expression in 2,394 patient specimens from different tumor entities. They observed the highest expression levels overall of nectin-4 in bladder (60%) and breast cancer (53%), in ovarian, head and neck and esophageal tumors the staining intensity was significantly lower [6]. In a study with patients with bladder cancer after treatment with platinum and anti programmed death 1/ programmed death ligand 1 therapy Nectin-4 expression was detected in all patient samples tested [7].

Enfortumab verdotin (EV), a Nectin-4-targeted antibody conjugated with the microtubule disrupting agent monomethyl auristatin E (MMAE), has demonstrated excellent response rates in locally advanced bladder cancer [8, 6, 9]. Approval of EV by the U.S. Food and Drug Administration (FDA) in a platinum and immune checkpoint inhibition refractory setting of bladder cancer was based on findings, which demonstrated objective response rates of 44% [10]. In 2020 EV combined with pembrolizumab was accepted by FDA as a firstline treatment for cisplatin-ineligible patients with locally advanced or metastatic urothelial carcinoma [11].

Although the vast majority of head and neck carcinomas are squamous cell carcinomas (HNSCC), they share some morphological and molecular homology with urothelial carcinoma, especially in their mutation profile [12]. HNSCC has a high mortality rate of 40% to 50% [13]. Major risk factors are tobacco use, alcohol consumption and infection with oncogenic human papillomavirus (HPV) [14]. Principal treatments are surgical resection of the primary tumor and neck dissection followed by a risk adapted adjuvant radiation or/ and platinum based chemotherapy or primary definitive chemoradiation [15]. So far cetuximab, a monoclonal antibody targeting epidermal growth factor receptor (EGFR) that has led to an increase of survival compared to conventional platinum based chemotherapy is the only successful targeted therapy approach in this disease [16]. Latest additions to treatment options have been agents targeting immune checkpoint receptors such as program death 1: they are currently approved for cisplatin refractory recurrent and or metastatic HNSCC [17–19]. However, only a minority of HNSCC patients show a clinical response to anti-PD-1 therapy. In a small cohort about a third of patients treated with anti-PD-L1/PD-1 agents showed hyperprogression, which correlated with shorter survival [20].

Therefore, further research to identify new and additional targets in HNSCC is urgently needed. In this study, we aimed to determine the rate of Nectin-4 positivity in a contemporary cohort of HNSCC and to correlate these findings with clinico-pathological parameters.

## RESULTS

### Patient characteristics

All patients were diagnosed with HNSCC at the Institute of Pathology of the University Hospital Bonn between 2004 and 2014. 16.6% were female, 83.4% were male. Mean patient age at diagnosis was 64.4 years (± 9.6 years SD). The majority of the patients presented with a pT2 tumor stage (40%), followed by pT3 (25%). About a half of the patients had lymph node metastases (48.5%). Most tumors had an intermediate differentiation (G2 56.5%, Table 1).

**Table 1:** Clinico-pathological characteristics and Nectin-4 expression

Bonn HNSCC cohort						
	Total	Nectin-4 negative	Low Nectin-4	Medium Nectin-4	High Nectin-4	*p*-value^*^
**All HNSCC cases**	*n* = 159	22 (13.8%)	85 (53.5%)	43 (27.0%)	9 (5.7%)	
**Patients with clinical data**	*n* = 151					
**Normal tissue**	*n* = 28	1 (3.6%)	8 (28.6%)	19 (67.9%)	0 (0%)	
**Gender**						
Male	126 (83.4%)	13	66	38	9	0.010^1^
Female	25 (16.6%)	5	17	3	9	
**Age [mean, standard deviation]**	64.4 [9.6]	65.27 [11.84]	64.16 [9.60]	64.45 [10.10]	64.17 [5.19]	
**Localization**						
Oral cavity	8 (7.8%)	1	5	0	2	0.992^2^
Oropharynx	59 (57.8%)	8	29	18	4	
Hypopharynx	7 (6.8%)	0	5	2	0	
Larynx	28 (27.2%)	2	17	9	0	
Unknown	49					
**Tobacco consumption**						
Non-smokers	6 (12.5%)	0	0	4	2	0.001^1^
Smokers	42 (87.5%)	2	27	13	0	
Unknown	111					
**Alcohol consumption**						
No alcohol	2 (4.5%)	0	2	0	0	0.067^2^
Occasional	28 (63.6%)	1	14	12	1	
Frequent	14 (31.8%)	1	9	3	1	
Unknown	115					
**HPV status (p16)**						
p16 negative	117 (73.6%)	19	67	26	5	0.005^1^
p16 positive	42 (26.4%)	3	18	17	4	
**T-stage**						
pTis	1 (0.7%)	0	1	0	0	0.444^2^
pT1	26 (18.6%)	2	17	5	2	
pT2	56 (40.0%)	7	27	19	3	
pT3	35 (25.0%)	6	22	5	2	
pT4	22 (15.7%)	2	11	8	1	
Unknown	19					
**Lymph node involvement**						
pN0	54 (41.9%)	9	29	14	2	0.580^2^
pN1	19 (14.7%)	1	10	8	0	
pN2	53 (41.1%)	7	28	13	5	
pN3	3 (2.3%)	0	3	0	0	
Unknown	29					
**Distant metastases**						
M0	70 (98.6%)	7	42	18	3	0.695^1^
M1	1 (1.4%)	0	1	0	0	
Unknown	88					
**Grading**						
G1	3 (2.2%)	0	1	2	0	0.545^2^
G2	78 (56.5%)	8	46	18	6	
G3	57 (41.3%)	8	29	17	3	
Unknown	21					

^1^Mann–Whitney *U* test. ^2^Kruskal-Wallis-test. ^*^all Nectin-4 expression categories (negative, low, medium and high) included.

### Expression patterns of Nectin-4 in HNSCC

Immunohistochemistry of Nectin-4 showed a cytoplasmic and membranous staining pattern in benign and malignant tissues.

**Figure 1:**
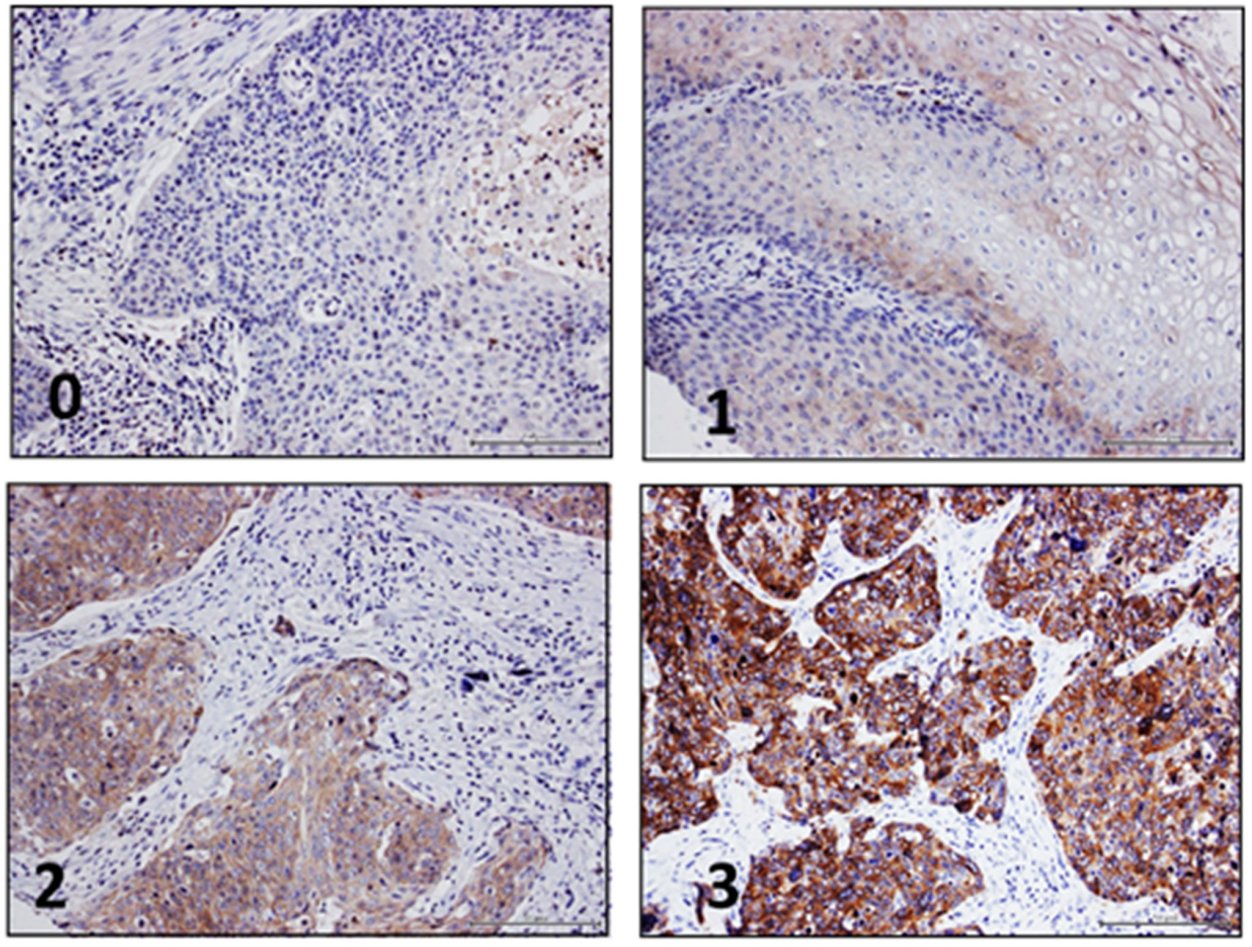
Nectin-4 immunohistochemistry in HNSCC. Representative images of HNSCC demonstrating negative (0), low (1), medium (2) and high (3) Nectin-4 protein levels.

Normal tissue was available of 28 patients, which showed low or medium expression of Nectin-4 in most cases (96.4%). In normal mucosa, Nectin-4 was mainly expressed in keratinocytes, with emphasis on basal layers, in which epidermal cells with proliferative ability reside. In HNSCC, 13.8% of cases were Nectin-4 negative, 53.5% showed a weak expression, 27.0% showed moderate levels and 5.7% show a high expression of Nectin-4 (Figure 1).

### Association between Nectin-4 expression and clinico-pathological parameters

In total, Nectin-4 protein expression was quantified in 159 tissue samples by immunohistochemistry (IHC). Clinical information including follow up data was available for 151 patients (Table 1).

Explorative statistics showed no associations of Nectin-4 expression with tumor stage, grade, occurrence of lymph node metastases, distant metastases, tumor localization, or patient age. Tumors of female patients and non-smokers showed significant higher rates of Nectin-4 expression than male patients or smokers (*p* = 0.010, *p* = 0.001, Table 1). Furthermore, p16 positive HNSCC also showed significantly higher Nectin-4 expression (*p* = 0.005, Table 1) and Nectin-4 expression was correlated with positive p16- status (*p* = 0.004, correlation coefficient 0.225).

**Figure 2:**
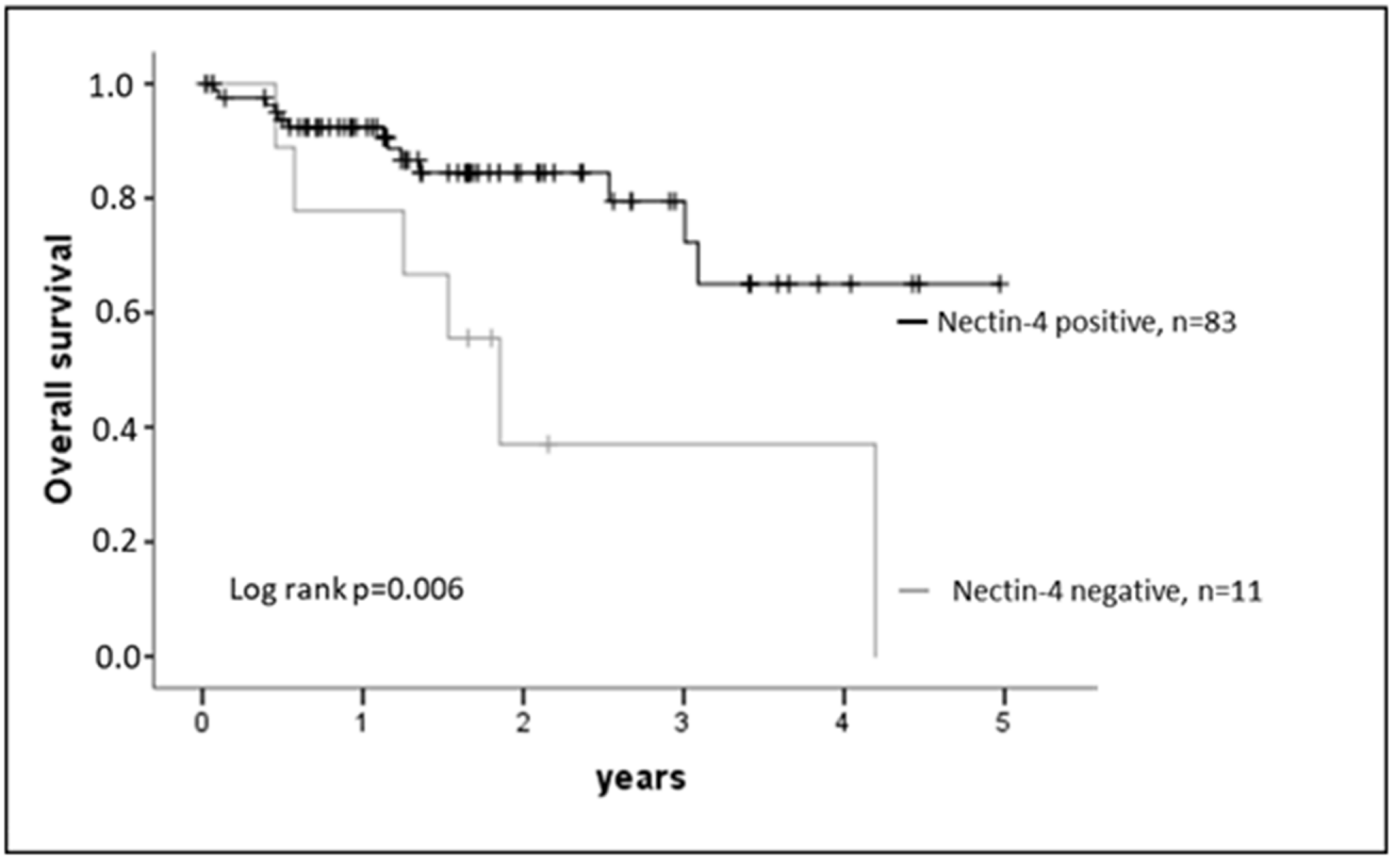
Nectin-4 expression and outcome. Nectin-4 expression correlates with overall survival of HNSCC patients. Log rank test *p* = 0.006.

A prognostic potential of Nectin-4 expression in HNSCC was indicated by Kaplan-Meier estimates (Figure 2): Nectin-4 positive tumors showed a significantly better survival (log rank test *p* = 0.006). In a multivariable Cox regression model Nectin-4 expression was also a significant marker for survival when adjusting for tumor stage, lymph node metastases and p16 (*p*-value model *p* = 0.006, Hazard rate Nectin-4 0.418, regression coefficient −0.969, *p* = 0.041; Table 2).

**Table 2:** Multivariate cox regression model (inclusion)

	Hazard rate [95% confidence intervals]	Regression coefficient	*p*-value	*p*-value model
pT	1.898 [1.119–3.219]	0.701	0.008	0.006
pN	1.623 [0.989–2.666]	0.391	0.053	
p16	0.521 [0.128–2.129]	−0.521	0.461	
Nectin-4	0.418 [0.166–1.055]	−0.969	0.041	

## DISCUSSION

Squamous cell carcinomas of the head and neck region are among the ten most commonly diagnosed malignancies in men in the Western world (Cancer Statistics, 2021). Still, the survival rate for HNSCC has improved only modestly over the past three decades [21] and new therapy options are needed. Nectin-4 is the target molecule of an antibody-drug conjugate (Enfortumab vedotin, EV) that has been FDA-approved for the treatment of metastatic urothelial carcinoma following progression on a platinum-containing chemotherapy and immune checkpoint blockade. Response rates to EV in this tumor type are encouraging (Rosenberg, 2019). This may be explained by the high expression rates of Nectin-4 in urothelial carcinomas. The positive clinical responses seen to EV therapy in eligible urothelial carcinoma cases encourage to identify further fields of application in other tumors.

Nectins are members of the immunoglobulin superfamily and play a role in E-cadherin-based cell adhesion of epithelia [22, 23].

Nectin-4 is relative widely expressed in solid tumors. In an immunohistochemistry based screening study, Chalitta-Eid et al. found positivity of Nectin-4 in bladder cancer (83%), and other solid cancers including HNSCC, which was positive in 59% of cases [6]. We found a slightly higher rate of Nectin-4 positivity, with only 13.7% negative cases, but this difference may be ascribed to different sensitivities of the immunohistochemistry assays. In our cohort Nectin-4 was mostly expressed at low or medium levels, which matches previous data [6].

To our knowledge we are the first to analyze Nectin-4 protein expression levels in HNSCC in correlation with clinico-pathological data. We did not observe an association of Nectin-4 expression with tumor stage, lymph node involvement or grading. This matches observations in other tumor entities (e.g., gastric cancer, ovarian cancer) [24, 25]. In gastric cancer high Nectin-4 expression was associated with higher tumor stage, lymph node metastases and poorer grading [26]. In breast cancer some studies link Nectin-4 expression with lower tumor stage and less lymph node involvement [27].

In this study, significant associations of Nectin-4 expression with gender, smoking and p16 status could be demonstrated. Higher Nectin-4 expression was found in the group of p16/ HPV positive HNSCC and non-smokers. This is of interest, as the mutational profile of HPV positive and negative HPV HNSCC differs significantly [28, 29]. HPV positive HNSCC show a better therapeutic response and survival [30, 31].

Several studies have linked Nectin-4 expression to disease progression. A poorer prognosis of patients with Nectin-4 positive tumors has been reported for bladder cancer [6] and esophageal cancer [32], whereas the data for breast cancer appears to be inconclusive so far [33, 27]. In this study, Nectin-4 positive HNSCC cases showed longer survival times. One may assume that this may be due to the positive, but weak correlation of Nectin-4 with p16, as p16 positive tumors have clinically a better prognosis. However, the multivariable analysis including p16 as a prognostic variable confirms an independent prognostic value of Nectin-4, which ought to be validated in larger cohorts.

The better prognosis of Nectin-4 positive HNSCC matches results of Tanaka et al.: In *in vitro* studies of cutaneous squamous cell carcinoma silencing of Nectin-4 lead to increased cell migration and inhibited proliferation [3]. In other tumor entities like ovarian cancer *in vitro* studies also suggest a link between Nectin-4 and proliferation and migration, but the exact effects were partly contradictory [34]. Another possible explanation for the better prognosis of Nectin-4 positive HNSCC could be due to the immune modulatory function of Nectin-4. Nectins are ligands of the inhibitory receptor T-cell immunoreceptor with Ig and ITIM domains (TIGIT), a inhibitory checkpoint receptor, which is expressed on most natural killer cells cells and multiple T cell subsets, including CD8+ tumor infiltrating lymphocytes (TILs) [4, 5]. Further studies are needed to evaluate the function of Nectin-4 in HNSCC.

In summary, this study confirms a high positivity rate of HNSCC for Nectin-4, which supports to explore its value as a target for EV therapy. It needs to be clarified in further studies, which cases qualify for this type of experimental therapy, as this is not without side effects [35].

## MATERIALS AND METHODS

### Patient population and tumor specimens

Evaluation of Nectin-4 protein expression was performed on tissue microarrays (TMA) constructed from a cohort of 159 clinically annotated HNSCC patients treated surgically with curative or palliative intent between 2011 and 2014 at the University Hospital Bonn as described previously [36]. The study was approved by the institutional review board of the University of Bonn (#148/11) and the ethic committee of the University Hospital Bonn.

### Immunohistochemistry

Immunohistochemical staining was conducted as described previously [36]. Nectin-4 antibody was used at a dilution of 1:1,000 (clone EPR15613-68, dilution 1:1,000, Abcam, Cambridge, UK). All supplementary reagents used for Nectin-4 staining were obtained Ventana Medical Systems. All tissue sections were counterstained with Mayer´s hematoxylin (Merck, Darmstadt, Germany). CS and JFL assessed Nectin-4 protein expression independently. Staining intensity was classified into four categories: no expression (0), low expression (1), medium expression (2) and high expression (3) (Figure 1).

### Statistics

Statistical analyses were carried out using the SPSS 25 software package (IBM, Armonk, NY). Comparisons between variable clinical pathological groups were made using the Mann–Whitney test and the Kruskal–Wallis test. Correlation analyses between variable groups were determined by Spearman’s rank correlation coefficient. Survival time was estimated by Kaplan-Meier analyses and compared among patient subsets using log-rank test, multivariate regression model was performed using Cox regression. All statistical tests were two-sided. *P*-values under 0.05 were considered significant.
